# Surface reconstructions govern ice nucleation on silver iodide

**DOI:** 10.1126/sciadv.aea2378

**Published:** 2025-10-31

**Authors:** Johanna I. Hütner, Andrea Conti, David Kugler, Franziska Sabath, Kim Noelle Dreier, Hans-Georg Stammler, Florian Mittendorfer, Angelika Kühnle, Michael Schmid, Ulrike Diebold, Jan Balajka

**Affiliations:** ^1^ Institute of Applied Physics, TU Wien, Vienna 1040, Austria.; ^2^ Department of Chemistry, Bielefeld University, Bielefeld 33615, Germany.

## Abstract

Silver iodide (AgI) is among the most effective ice-nucleating agents, attributed to its close lattice match with hexagonal ice. However, the atomic-level mechanism behind its efficiency remains unclear. The basal surfaces of AgI are polar and inherently unstable, necessitating a compensation mechanism, such as surface reconstruction, which may disrupt the favorable lattice match with ice. We combine noncontact atomic force microscopy with advanced computational modeling to determine the atomic structure of basal AgI surfaces in ultrahigh vacuum. The Ag-terminated (0001) surface exhibits a (2 × 2) reconstruction with ordered Ag vacancies, preserving a hexagonal arrangement of surface atoms that facilitates epitaxial ice growth. In contrast, the I-terminated (000$\bar{1}$) surface adopts a complex rectangular reconstruction, incompatible with continuous ice layer formation. These findings highlight the decisive role of surface atomic structure and indicate that the Ag-terminated basal plane is primarily responsible for efficient ice nucleation on AgI.

## Teaser

Atomic-scale imaging of polar AgI surfaces reveals why only the silver-terminated basal face promotes epitaxial ice formation.

## INTRODUCTION

Freezing of water, a phase transition that affects all life on Earth, is typically initiated by heterogeneous ice nucleation on solid particles. While homogeneous ice nucleation in pure water to ice requires temperatures as low as −38°C, silver iodide (AgI) can trigger ice formation already at −4°C (*1*). This remarkable ice-nucleating efficiency has been attributed to the near-perfect lattice match (less than 2%) between the AgI(0001) and hexagonal ice I_h_(0001) basal planes (*2*–*4*). However, the lattice match alone is insufficient to explain the remarkable ice-nucleating ability, as pointed out earlier (*5*). Despite extensive research on cloud seeding for precipitation control (*6*–*11*), atmospheric field studies cannot capture detailed, atomic-level mechanisms governing ice nucleation on AgI.

The basal planes of AgI, although structurally well matched to ice I_h_, are polar and inherently unstable surfaces. The wurtzite structure of β-AgI consists of alternating planes of positively charged Ag^+^ and negatively charged I^−^ ions (fig. S1). A cut perpendicular to the *c* axis creates two opposite surfaces, each terminated by only one ion type. This charge arrangement results in an electrical dipole moment perpendicular to the surface, causing a diverging electrostatic surface energy and renders the basal surfaces unstable [Tasker type III; (*12*)]. To compensate for this instability, the surface charge density must be modified, which can be achieved by altering the surface stoichiometry through the formation of a surface reconstruction (*13*, *14*). However, such modifications may compromise the structural similarity with hexagonal ice. To date, the stabilization mechanism of AgI surfaces remains unknown. Experiments in solution suggest that the required surface charge imbalance may be achieved via ion adsorption (*2*), whereas pure water is considered unable to provide the necessary charge compensation (*15*). Thus, clean surfaces free of charged adsorbates can be stabilized only by changing their stoichiometry or oxidation state.

Experimental studies on AgI surfaces have been hindered by the material’s insulating nature, photosensitivity, and the scarcity of suitable single crystals. In solution, imaging is further complicated by hydration layers that can obscure the bare surface (*16*). Here, we use noncontact atomic force microscopy (nc-AFM) based on the qPlus force sensor (*17*) to image the atomic structure of the basal planes of solution-synthesized AgI crystals (*2*, *18*) cleaved in ultrahigh vacuum (UHV). The qPlus sensor operates without optical readout, thereby avoiding photolytic degradation of AgI during measurement, and is well suited for imaging electrically insulating materials. Notably, qPlus-based nc-AFM has previously been used to resolve weakly adsorbed hydrogen-bonded water networks (*19*–*21*) and bulk-like ice surfaces (*22*), making it particularly suitable for imaging ice on insulating AgI surfaces.

Atomically resolved images obtained in this work reveal that the two basal planes of AgI undergo distinct surface reconstructions to compensate polarity, leading to fundamentally different modes of ice growth from the vapor phase. The Ag-terminated surface promotes epitaxial ice growth, forming a two-dimensional hexagonal ice network. In contrast, the I-terminated surface lacks an epitaxial relationship with ice I_h_, resulting in the formation of isolated three-dimensional ice clusters. These findings demonstrate the direct role of atomic surface structure in heterogeneous ice nucleation.

## RESULTS

### Ag-terminated surface forms (2 × 2) Ag-vacancy reconstruction

**Fig. 1.**
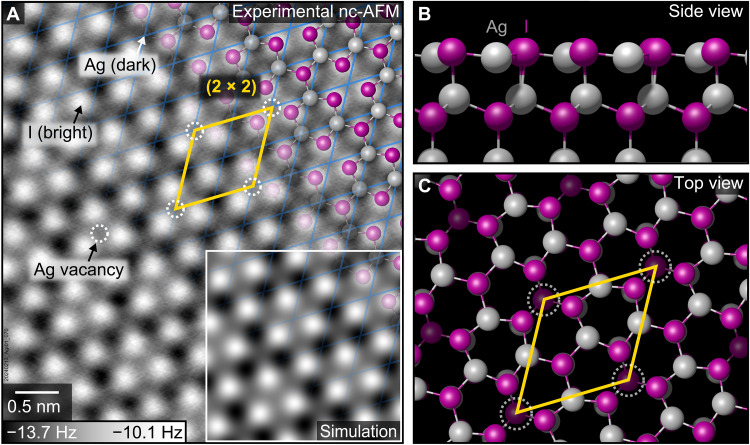
The Ag-terminated AgI(0001) surface exhibits a (2 × 2) reconstruction. (**A**) nc-AFM image of a surface cleaved in UHV at 100 K, acquired with an I-terminated tip. Iodine ions appear as bright (repulsive to the nc-AFM tip) and Ag ions as dark (attractive). The image was acquired at 77 K with an oscillation amplitude of 100 pm and a sample bias of +1 V. The blue grid indicates the (1 × 1) bulk periodicity. (**B** and **C**) Structure model of the reconstructed surface. The Ag vacancies (every fourth surface Ag site) are marked with white dashed circles. The inset in (A) shows a simulated nc-AFM image of the computationally relaxed structure in (B) and (C).

The Ag-terminated AgI(0001) surface cleaved in UHV at 100 K adopted a (2 × 2) reconstruction with one-quarter of the surface Ag sites vacant (Fig. 1A). The surface structure of neighboring terraces, separated by ~0.38-nm steps (half the unit cell height), was rotated by 180°, consistent with the wurtzite structure. The (2 × 2) reconstructed surface remained unchanged upon warming the sample to room temperature. On the basis of the experimental images, a structure model of the (2 × 2) reconstruction was created by removing one-fourth of the surface Ag atoms from a bulk-truncated AgI surface (Fig. 1, B and C). In the computationally optimized model, the remaining surface silver atoms relaxed inward, below the iodine plane, to avoid an undercoordinated position above the surface (Fig. 1B) (*23*). The surface I atoms surrounding Ag vacancies relaxed slightly toward the vacancy sites causing lateral displacement of neighboring Ag atoms to preserve Ag─I bond lengths (fig. S2). To visually verify the computationally optimized structure, we generated a simulated nc-AFM image (Fig. 1A, inset), showing excellent agreement with the experimental image. Despite being located beneath the surface iodine plane, the undercoordinated surface Ag^+^ ions yield a strong attractive contrast, observed as dark frequency minima in both experimental and simulated images. The inward relaxation of surface Ag atoms enables resolving both Ag and I sublattices simultaneously in nc-AFM (fig. S3).

### Epitaxial ice growth on the reconstructed Ag-terminated surface

**Fig. 2.**
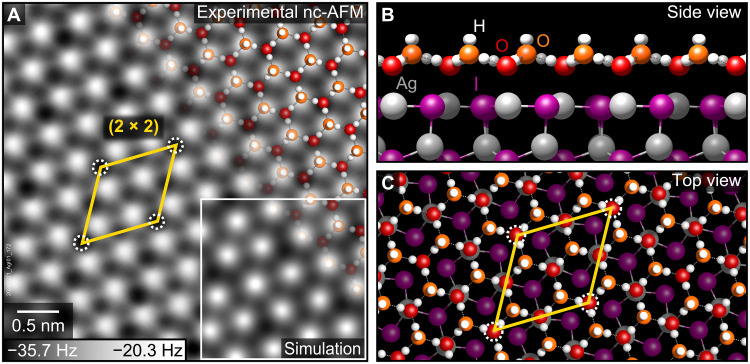
Epitaxial ice layer on the reconstructed Ag-terminated AgI(0001) surface. (**A**) nc-AFM of the ice layer on the Ag-terminated surface exposed to 2.5 L of H_2_O vapor at 102 K, recorded at 77 K with 100 pm oscillation amplitude and 0 V sample bias. The yellow rhombus indicates the (2 × 2) periodicity of the reconstructed AgI substrate, and the white dashed circles mark the positions of the Ag vacancies. (**B** and **C**) Structure model of the epitaxial ice layer on the reconstructed AgI(0001) surface. The inset in (A) shows a simulated nc-AFM image of the structure in (B) and (C) assuming a H^+^-terminated tip.

Ice formed a continuous hexagonal network on the (2 × 2)-reconstructed AgI(0001) surface (Fig. 2A) upon exposure to 2.5 Langmuir (L) of H_2_O vapor at 102 K (1 L = 1.33 × 10^−6^ mbar·s). The applied nominal dose slightly exceeds the calculated monolayer dose of 2.3 L, corresponding to two water molecules per (1 × 1) cell (one H_2_O molecule per each Ag and I site). Figure 2 (B and C) shows the computationally optimized, lowest-energy structure of the ice layer, containing water molecules in two orientations. The lower, nearly horizontal H_2_O molecules (red in Fig. 2) form a hexagonal array directly above the surface Ag sites. The higher, nearly vertical H_2_O molecules (orange) reside above the hollow sites of the AgI(0001) surface. In accordance with the ice rules (*24*), each oxygen atom of the upper water molecules accepts two hydrogen bonds from its lower (horizontal) H_2_O neighbors. The horizontal water molecules accept only one hydrogen bond from a neighboring upper (vertical) H_2_O, but ¾ of them also form a weak additional bond between the O atom and the surface Ag atom below. The water molecules atop the Ag vacancies lack the bond to the substrate and are stabilized laterally by the surrounding H_2_O molecules. The upward-pointing hydrogen atoms of the upper H_2_O molecules can form hydrogen bonds to the subsequent layer, potentially promoting further ice growth in the vertical dimension.

The bright contrast in Fig. 2A is attributed to the repulsion between the upright hydrogen atoms and the (presumably H^+^-terminated) tip. The absence of O─Ag coordination of the H_2_O molecules above the Ag vacancy sites leads to increased attraction to the positively charged tip and causes a modulation of the dark minima in Fig. 2A with the (2 × 2) periodicity of the reconstructed AgI substrate.

Both horizontal and vertical water molecules adopt three in-plane orientations, rotated by 120°, present in equal populations. This threefold symmetry results in a net zero lateral dipole moment but renders adjacent ice hexagons formally inequivalent, which, assuming long-range order (*25*), leads to a (3 × 3) periodicity in the ice layer. The smallest repeat unit is thus a (6 × 6) unit cell containing 72 H_2_O molecules, the least common multiple of the (3 × 3) periodicity of the ice layer and the (2 × 2) reconstruction of the AgI(0001) substrate. In the presence of the ice layer, the surface Ag atoms in the computationally optimized model relaxed upward from their positions on the bare surface to a position slightly above the iodine plane.

### Rectangular reconstruction of I-terminated surface prevents epitaxial ice growth

**Fig. 3.**
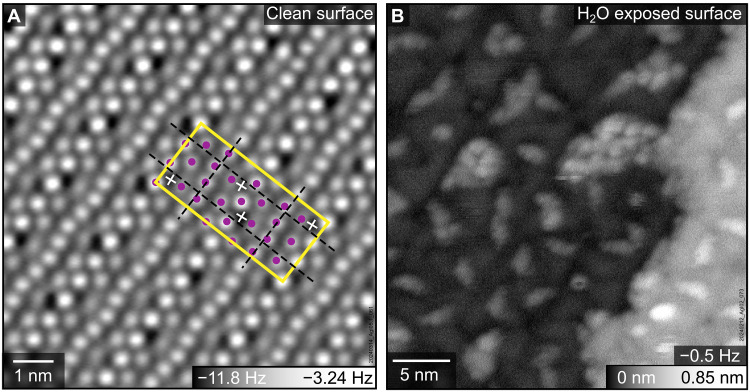
The (4 × 5√3)_rect._ reconstruction of the I-terminated AgI(000$\bar{1}$) surface: As cleaved and exposed to water vapor. (**A**) nc-AFM image of the cleaved, I-terminated surface acquired at 10 K with an oscillation amplitude of 100 pm and a sample bias of 0 V. The (4 × 5√3)_rect._ unit cell is marked with a yellow rectangle; glide planes are indicated with dashed black lines. Each reconstruction unit cell contains 26 I atoms (imaged as bright, highlighted purple) and four additional species discussed in the text (imaged as dark, marked with white crosses). (**B**) Topography image (constant frequency shift) of ice clusters on the surface exposed to 0.25 L H_2_O at 99 K. The image was recorded at 77 K with an amplitude of 300 pm and a sample bias of 0 V.

The I-terminated basal plane of the AgI crystals cleaved in UHV at room temperature exhibited a complex surface reconstruction with rectangular symmetry (Fig. 3A). The reconstruction unit cell (1.8 nm by 4.0 nm) extends over four lengths of the bulk unit vector **a** in one direction and 10 bulk unit vector lengths projected to an orthogonal direction ^√3^/_2_ **a**, yielding a (4 × 5√3)_rect._ reconstruction. This reconstruction unit cell exhibits twofold rotational symmetry and glide-plane symmetry along both axes, with glide axes located at ¼ and ¾ of the unit cell length in both directions (Fig. 3A, black dashed lines). The *pgg* glide-plane symmetry causes extinction of odd fractional spots along both axes in the fast Fourier transform (FFT) of nc-AFM images (fig. S4). The (4 × 5√3)_rect._ reconstructed surface exhibits translational domains and three rotational domains rotated by 120°, coexistent within a single terrace. The translational domains are laterally shifted at the center of the reconstruction unit cell (fig. S4A), giving rise to antiphase domain spot splitting in the FFT (fig. S4B).

Upon exposure to 0.25 L of H_2_O at 99 K, the reconstructed I-terminated AgI(000$\bar{1}$) surface did not support the formation of a continuous ice layer. Instead, isolated three-dimensional ice clusters were observed on the surface (Fig. 3B).

## DISCUSSION

The bulk-terminated (0001) and (000$\bar{1}$) planes require an imbalance of ±¼ of surface charge density, to avoid a macroscopic dipole moment perpendicular to the surface (*12*–*14*). To meet this nonpolarity requirement, both basal planes reconstruct, although the atomic details of each reconstruction differ substantially. On the Ag-terminated (0001) surface, the required charge density imbalance is established through vacancies at every fourth Ag site of the (2 × 2) reconstruction. In contrast, the I-terminated (000$\bar{1}$) surface exhibits a (4 × 5√3)_rect._ reconstruction. The area of this reconstruction cell is equivalent to that of a (4 × 10) cell of the stoichiometric bulk-truncated surface, containing 40 Ag and 40 I atoms. Achieving a ¼ deficit of negative charge would require reducing the number of I atoms per unit cell to 30. However, nc-AFM imaging (Fig. 3A) shows that the (4 × 5√3)_rect._ reconstruction cell contains only 26 I atoms. Thus, the reconstructed surface must provide four additional negative charges to achieve the required charge deficit. The four dark features observed in each reconstruction unit cell (white crosses in Fig. 3A) are likely candidates to balance the charge of the missing surface I atoms. Although nc-AFM cannot directly identify these species, possible explanations include additional I atoms or Ag vacancies in the subsurface layer.

The atomic structure of AgI basal planes plays a crucial role in ice growth. The hexagonal symmetry of the (2 × 2) reconstructed Ag-terminated surface remains compatible with epitaxial ice, and the ordered Ag vacancies do not hinder the formation of a continuous ice layer, consistent with previous computational studies showing that isolated Ag defects have no impact on ice nucleation rates (*26*). The ordered ice network observed in Fig. 2 and the absence of amorphous ice or clusters indicate that ice growth on AgI(0001) at 100 K is not kinetically limited. The subtle (2 × 2) height modulations within the epitaxial ice layer may be difficult to resolve under the more challenging conditions in liquid, leading to the apparent (1 × 1) periodicity reported previously (*2*). With upward-pointing hydrogen atoms, the ice layer on AgI(0001) acts as a seed layer and may facilitate vertical ice growth, unlike the hydrophobic water monolayer reported on Pt(111) (*27*, *28*). In contrast, the rectangular symmetry and larger I–I spacing (≈6 Å in most regions) of the I-terminated surface disrupt epitaxial alignment, preventing the growth of a continuous ice layer and favoring the formation of three-dimensional clusters.

The observed asymmetry in ice nucleation efficiency between the two basal planes has previously been modeled using polar unreconstructed surfaces (*5*, *29*–*31*) and experimentally attributed to the opposite polarities of the pyroelectric surface charge developed upon cooling (*32*). However, our results show that the dipole moment of the AgI basal planes is compensated by surface reconstructions and that efficient ice nucleation on the Ag-terminated side is facilitated by its epitaxial alignment with ice. These findings support the proposed necessity of an epitaxial relationship between AgI and hexagonal ice for efficient ice nucleation (*31*).

AgI presents substantial challenges for computational modeling (*4*). Density functional theory (DFT) calculations showed a strong dependence on the choice of functional and often failed to reliably reproduce even the experimental lattice parameters of AgI bulk (fig. S5). In our work, a key criterion for assessing the correctness of proposed structure models was the visual agreement between simulated and experimental AFM images. The simulated image contrast of the (2 × 2) reconstructed Ag-terminated surface is largely determined by the relaxation of the surface atoms, particularly the inward relaxation of Ag atoms, which varied by up to 0.4 Å among different DFT functionals using the same input structure (fig. S5). Both Ag and I are highly polarizable and require a correct treatment of van der Waals interactions, which are not accurately described by standard DFT. In contrast, random phase approximation (RPA) calculations yielded excellent agreement with the bulk lattice geometry (fig. S5) and provided a stable model of the reconstructed AgI(0001) surface, closely matching the nc-AFM data (Fig. 1). However, the high computational cost of RPA calculations limits their applicability to small computational slabs. The computationally inexpensive Perdew-Burke-Ernzerhof exchange-correlation functional with the dispersion correction D3 (PBE-D3) provided good agreement with RPA results for the reconstructed AgI(0001) surface and produced simulated AFM images in excellent agreement with experiment (fig. S5). The (6 × 6) computational slab of the ice layer (Fig. 2), optimized with PBE-D3, quantitatively reproduced the upward relaxation of surface Ag atoms upon water adsorption, as observed with RPA on a smaller (2 × 2) slab. Thus, the PBE-D3 functional provides an efficient and sufficiently accurate alternative to RPA calculations for modeling ice on AgI.

Previous computational studies of ice nucleation on AgI typically assumed bulk-truncated substrates, often with fixed atomic positions to circumvent the inherent instability of polar slabs (*5*, *26*, *29*–*31*, *33*–*39*). These approaches may lead to inaccurate conclusions as they rely on an unstable support and do not account for the relaxations of the AgI surface in response to the ice layer. Our findings demonstrate the critical role of the AgI substrate and its detailed atomic structure in the ice nucleation process and the necessity to treat the AgI + ice system as a whole to obtain accurate computational predictions.

In summary, this work provides fundamental mechanistic insights into the stabilization of polar AgI surfaces in UHV and elucidates how AgI serves as an effective ice nucleus, despite deviating from its bulk-truncated structure. While the environmental conditions in the cloud may play a role, the distinctly different behavior of Ag- and I-terminated surfaces demonstrates the importance of surface atomic structure in heterogeneous ice nucleation, with implications for atmospheric science and the development of ice-nucleating materials.

## MATERIALS AND METHODS

### Synthesis of AgI crystals and SC-XRD analysis

AgI was synthesized by precipitation from solution in the form of small pyramidal crystals with a hexagonal base (*2*, *18*). A cleaved tip of a pyramidal AgI crystal was glued to a glass capillary and examined with a Rigaku Supernova diffractometer using Mo Kα radiation (λ = 0.71073 Å). The crystal was kept at room temperature during data collection. Crystal data, in accordance with (*40*), are as follows: hexagonal symmetry, space group P6_3_mc (no. 186), lattice parameters *a* = 4.59637(17) Å, *c* = 7.5176(3) Å, unit cell volume 137.544(9) Å^3^, sum formulae (AgI) per unit cell *Z* = 2, absorption coefficient μ(Mo Kα) = 18.160 mm^−1^, and calculated density ρ_calc_ = 5.669 g/cm^3^. A total of 11,208 reflections were collected in the 2Θ range of 10.2° to 78.0°; 343 unique reflections (*R*_int_ = 0.0618, *R*_sigma_ = 0.0133) were used in all calculations. The structure was solved using Olex2 (*41*) with the SHELXT (*42*) structure solution program using Intrinsic Phasing and refined with the olex2.refine (*43*) package using Gauss-Newton minimization. The final *R* factor *R*_1_ was 0.0397 for 329 reflections with *I* ≥ 2*u*(*I*), and the final *wR*_2_ was 0.1011 for all data. The orientation of the polar axis is confirmed by a Flack parameter (*44*) of 0.01(3). On the basis of the single-crystal x-ray diffraction (SC-XRD) analysis, the base plane of the pyramid exposes the I-terminated (000$\bar{1}$) surface, while the cleavage plane oriented in the opposite direction is the Ag-terminated (0001) surface (fig. S6), consistent with the crystal habit observed in other wurtzite compounds, such as ZnO (*45*). Supplementary crystallographic data can be found in CCDC 2428830 and are available free of charge from the Cambridge Crystallographic Data Centre via https://www.ccdc.cam.ac.uk/structures.

### Sample preparation

For experiments on the Ag-terminated surface, the base of a pyramidal AgI crystal was glued to an Omicron-style stainless steel sample plate, and a stainless-steel stud was glued on top of the crystal using a UHV-compatible epoxy (EPO-TEK T7110) cured at 100°C for 90 min. For experiments on the I-terminated surface, a crystal was glued upside down. AgI photolytically decomposes when irradiated by light below 440 nm, as evidenced by a visible color change (*2*). To avoid light-induced degradation, the crystals were kept in the dark during the synthesis, manipulation, and measurements, only using red light when necessary. The AgI crystals were cleaved in the preparation chamber of a UHV system (base pressure of 1 × 10^−10^ mbar) by laterally pushing onto the stud glued to the crystal. Cleaving at room temperature and at ~100 K resulted in large, atomically flat terraces.

Cleaved samples were exposed to water vapor in the preparation chamber. Ultrapure H_2_O (Milli-Q, Millipore; 18.2 megohm·cm, ≤3 parts per billion of total organic carbon) was further cleaned by several freeze-pump-thaw cycles before dosing. The sample was inserted into a precooled manipulator and kept at ~100 K. Water vapor was introduced into the chamber at pressures of 1.3 × 10^−9^ or 1.3 × 10^−8^ mbar, maintained for 250 s to reach exposures of 0.25 L (1 L = 1 × 10^−6^ torr·s) or 2.5 L, respectively.

### Noncontact AFM measurements

nc-AFM measurements were conducted in the analysis chamber of the same UHV system (base pressure of 6 × 10^−12^ mbar). The cleaved samples were imaged with a Tribus LT-STM/AFM from Scienta Omicron equipped with a cryogenic preamplifier (*46*) using a qPlus tuning fork force sensor (*17*) (resonance frequency *f*_0_ = 22.7 kHz, quality factor *Q*_LN2_ ≈ 7800, *Q*_LHe_ ≈ 20,300, and force constant *k* = 1800 N/m) with an electrochemically etched tungsten tip attached to the oscillating prong. An atomically sharp tip apex was prepared by voltage pulses and tip indentations on a Cu(110) surface. Optionally, the metallic tip was functionalized by indentations into the clean AgI surface to obtain a negative, I-terminated tip, or into the ice-covered AgI surface yielding a positive tip, presumably H^+^-terminated, as indicated in the figure captions. The nc-AFM images were recorded at constant height unless specified otherwise. A bias voltage was applied to minimize the local contact potential difference and thereby the electrostatic forces between the tip and the sample. The nc-AFM images were undistorted to compensate for piezo creep (*47*).

### Computational modeling

The calculations were performed using the projector-augmented wave method (*48*, *49*) as implemented in the Vienna Ab-initio Simulation Package (version 6.4.3) (*50*), using either the semilocal PBE-D3 (*51*) or the adiabatic connection fluctuation dissipation theorem (ACFDT) in the random phase approximation (RPA) (*52*). The ACFDT-RPA ground-state energy was the sum of the ACFDT-RPA correlation energy and the Hartree-Fock energy, evaluated non–self-consistently using Kohn-Sham orbitals computed within DFT. For the RPA calculations, the bulk unit cell was optimized by gradually varying the in-plane and out-of-plane lattice parameters and then fitting the values of the total energy using the Birch-Murnaghan isothermal equation of state. The RPA calculations provided the most accurate description of the AgI bulk structure, with lattice parameters deviating by less than 0.1% from the experimental values (*53*), and were used for computational optimization of the bare, reconstructed AgI(0001) surface. Gaussian smearing with a width of 0.1 eV and 12 imaginary frequency points were used in the RPA calculations. The bulk structure was calculated with a cutoff energy of 800 eV, using a 6 × 6 × 4 *k*-point mesh to integrate the Brillouin zone. A lower cutoff energy of 400 eV was applied for the surface calculations with a 3 × 3 × 1 *k*-point mesh for the (2 × 2) unit cell. Larger computational slabs of epitaxial ice on the AgI(0001) surface were optimized with DFT using the PBE-D3 functional, which yielded good agreement with the lattice parameters of AgI, deviating by less than 0.5% from the experiment (*53*) at a substantially lower computational cost. The PBE-D3 functional contains corrections for an accurate description of van der Waals interactions in the ice layer (*54*) and was found to yield results comparable to the RPA calculations (the length of O─Ag bonds was 2.76 Å with RPA and 2.79 Å with PBE-D3) using a smaller computational test slab of an ice layer on the AgI surface. The geometry was optimized using the conjugate gradient method for the PBE-D3 calculations and in steps for the RPA calculations until the residual forces on the atoms were below 0.02 eV/Å.

The structure model of the (2 × 2)-reconstructed Ag-terminated surface was created such that the top surface contains one Ag vacancy and the bottom surface contains one I vacancy per (2 × 2) unit cell (in-plane dimensions: 9.18 Å by 9.18 Å), resulting in nonpolar surfaces. The computational slab contained six AgI bilayers with a total of 46 ions (23 Ag and 23 I). Periodically repeated slabs were separated by 12 Å of vacuum. Dipole corrections were applied, although their use did not affect the calculation results because the electric field in the vacuum gap was negligible (<0.1 V/nm). No conclusive calculations of the I-terminated slab have yet been performed due to the complexity of its reconstruction; these will be left for future work. The model of the epitaxial ice layer on the relaxed, Ag-terminated AgI(0001) surface consisted of 72 water molecules in a (6 × 6) unit cell (in-plane dimensions: 27.5 Å by 27.5 Å), the least common multiple of the (3 × 3)-periodic hexagonal ice layer and the (2 × 2) reconstructed AgI substrate. The epitaxial ice structure was optimized using the PBE-D3 functional with a 400 eV cutoff energy and a single *k*-point (Γ) for the large (6 × 6) cell.

### Simulations of AFM images

nc-AFM images of the relaxed models were simulated with the probe-particle model (PPM) (*55*, *56*), which includes the electrostatic potential above the surface (from the DFT calculation), Lennard-Jones potentials, and the elastic properties of the tip. The parameters of a negatively charged tip from (*57*) (*k*_*x*,*y*_ = 161.9 N/m, *k_z_* = 271.1 N/m, effective tip charge of −0.05 e) were used to simulate images of the clean surface. The standard atom radii used by the PPM did not reproduce the experimentally observed repulsive interaction between the tip and the surface I atoms. To account for the ionic character of surface iodine, the I radius was scaled by a factor of 1.06 (*r*_Pauling_/*r*_VdW_), the ratio of the Pauling radius of an I^−^ ion (*58*, *59*) and the van der Waals radius [half of the distance between I atoms in an I_2_ molecule; (*60*)]. With this modification, the simulated AFM images accurately represented the observed Pauli repulsion between an I-terminated tip and the I atoms of the surface. The experimental oscillation amplitude of 100 pm was used in the simulations. As the exact height of the tip in the experiment is not known, the height yielding the best visual agreement between the experiment and simulation was chosen. nc-AFM images of the ice layer were simulated with a positively charged tip (effective tip charge of +0.05 e) and elastic parameters *k*_*x*,*y*_ = 0.25 N/m, *k_z_* = 271.1 N/m, which was effective in modeling nc-AFM images of thick ice in (*22*).

## Supplementary Materials

### The PDF file includes:

Figs. S1 to S6 Legends for data S1 to S3 SC-XRD checkCIF/PLATON report
