# Supplementary material for: Surface reconstructions govern ice nucleation on silver iodide
Source: Sci Adv. 2025 Oct 31;11(44):eaea2378. doi: 10.1126/sciadv.aea2378 (PMC12577688; doi:10.1126/sciadv.aea2378)

Science Advances
AAAS

Supplementary Materials for

Surface reconstructions govern ice nucleation on silver iodide

Johanna I. Hütner *et al.*

Corresponding author: Jan Balajka, jan.balajka@tuwien.ac.at

Sci. Adv. **11**, eaea2378 (2025)
DOI: 10.1126/sciadv.aea2378

The PDF file includes:

Figs. S1 to S6
Legends for data S1 to S3
SC-XRD checkCIF/PLATON report

Other Supplementary Material for this manuscript includes the following:

Data S1 to S3

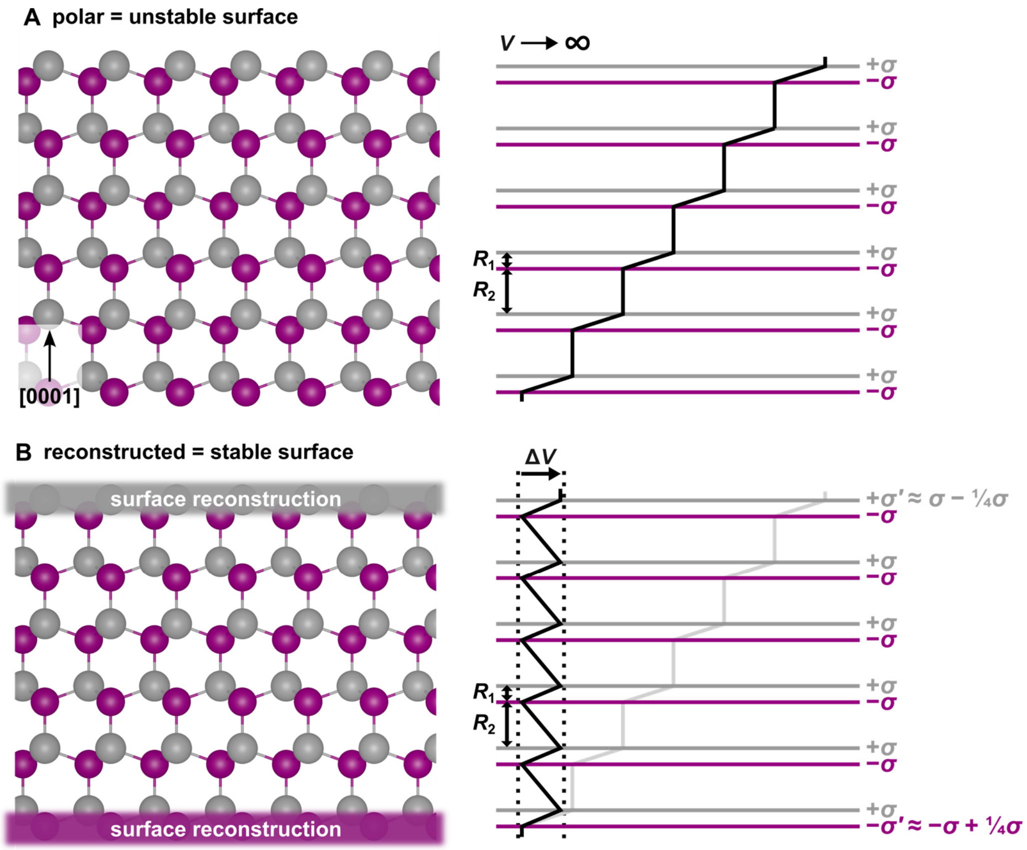

Fig. S1. Surface reconstructions stabilize polar AgI basal surfaces. β-AgI (wurtzite structure) consists of alternating planes of positive (Ag^+, gray) and negative (I^-, purple) ions stacked along the [0001] direction. (**A**) A bulk-terminated surface carries a net dipole moment perpendicular to the surface, leading to a diverging electrostatic potential V (analogous to a series of parallel-plate capacitors). This polar termination is intrinsically unstable (Tasker type III) and requires a compensation mechanism (*12*). (**B**) Modified charge density of the reconstructed surface provides a compensating electric field. The required surface charge density σ' is determined by the distances between successive Ag^+ and I^- planes in bulk β-AgI (R_1 = 95 pm and R_2 = 281 pm, Supplementary Data S3):

$$\sigma' = \frac{R_2}{R_1 + R_2}\sigma \approx 0.75\ \sigma,$$

where σ is the area charge density of the bulk-terminated surface (*13*). The (2 × 2)-ordered silver vacancies supply the necessary deficit of ¼ σ, rendering the reconstructed AgI(0001) surface non-polar and stable.

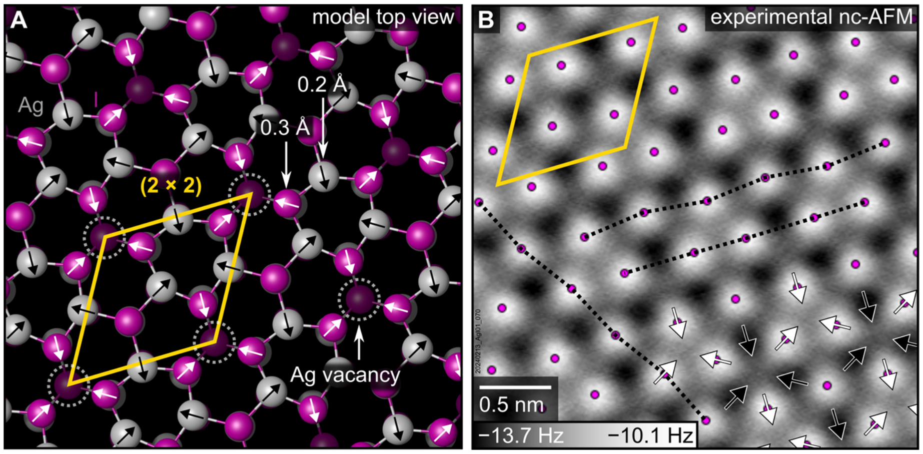

Fig. S2. Lateral relaxations of Ag and I atoms of the (2 × 2) reconstructed AgI(0001) surface. (**A**) Top view of the reconstructed (2 × 2) surface model. Arrows indicate the directions of the in-plane displacements of the surface Ag (0.2 Å) and I (0.3 Å) atoms from a perfect hexagonal pattern. Ag vacancies are marked with white dashed circles. These small shifts are discernible in the nc-AFM image in (**B**) and in the simulated AFM image (inset of Fig. 1A in the main text). Magenta dots mark the bright (repulsive) I atoms, and black dotted lines connecting the I atoms highlight the subtle lateral deviations.

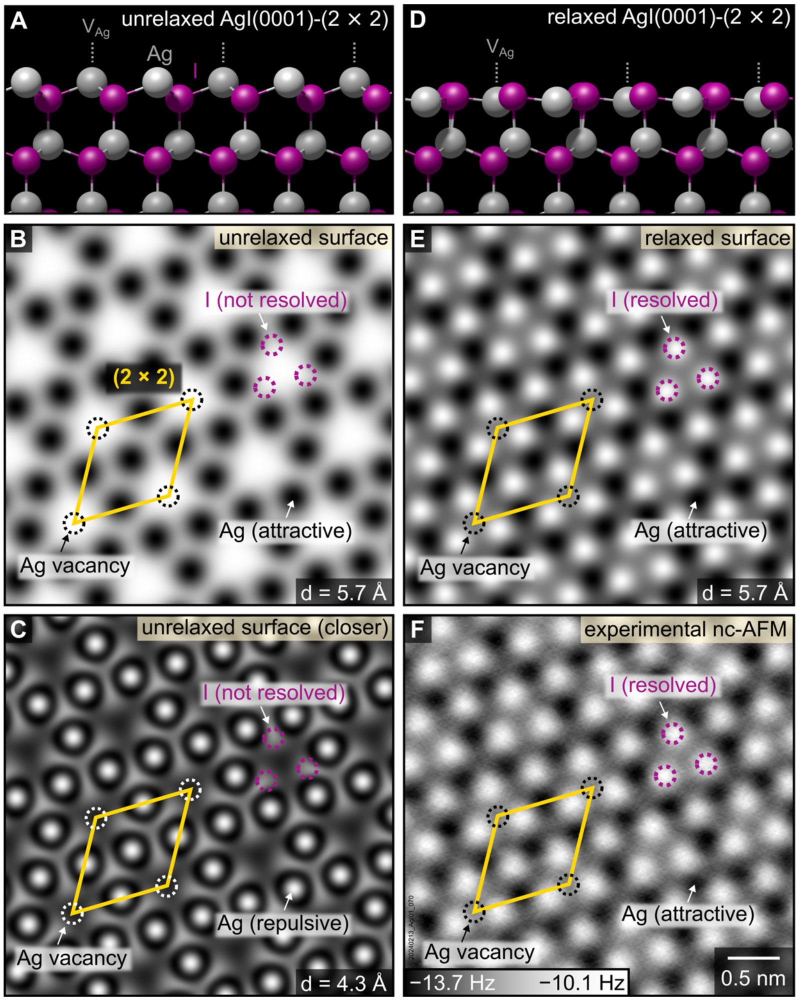

Fig. S3. Surface Ag inward relaxation enables resolving both Ag and I sublattices in nc-AFM. (**A, D**) Side views of the (2 × 2)-reconstructed AgI(0001) surface before and after relaxation. Ag vacancies are indicated with dotted lines. (**B, C**) AFM simulations of the unrelaxed model in (A) at different tip–sample distances. At a larger distance (B, 5.7 Å), surface Ag atoms appear attractive (dark). At a closer distance (C, 4.3 Å), the same Ag atoms are imaged as repulsive (bright) due to Pauli repulsion. In both cases, surface I atoms are not resolved. (**E**) AFM simulation of the relaxed surface shown in (D) at the same tip–sample distance of 5.7 Å shows resolution of both sublattices, with Ag appearing attractive, and I repulsive. (**F**) Experimental nc-AFM image of the (2 × 2) reconstructed AgI(0001) surface closely matching the simulated image in (E).

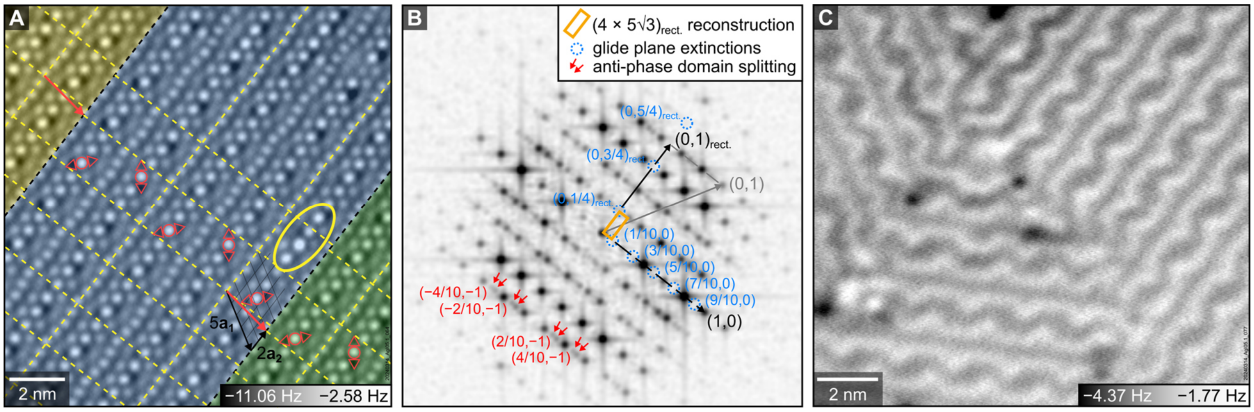

Fig. S4. I-terminated AgI(000$\bar{1}$) surface with glide-plane symmetry forms translational and rotational domains. (**A**) Nc-AFM image obtained in constant height at 10 K (amplitude 100 pm, sample bias 0 V). Iodine ions appear as bright/repulsive and silver ions as dark/attractive to the nc-AFM tip. The $(4 \times 5\sqrt{3})_{\mathrm{rect.}}$ periodicity of the reconstruction unit cell is highlighted with yellow dashed lines. The real-space unit cell with *pgg* plane-group symmetry is described in matrix notation by $\begin{pmatrix} 4 & 0 \\ -5 & 10 \end{pmatrix}$. A defective region is marked with a yellow ellipse. The four dark features in each unit cell are marked with red triangles and the bright I atom between adjacent triangles is marked with a red circle to highlight the glide-plane symmetry. The reconstruction forms translational domains shaded yellow, blue and green. The red arrow indicates the translation vector at the anti-phase domain boundary (black dashed lines) and is expressed in the basis of bulk unit vectors. (**B**) Fast Fourier transform (FFT) of the nc-AFM image in (A). The reciprocal reconstruction cell is marked with an orange rectangle. The vectors (1,0) (black) and (0,1) (gray) mark the hexagonal bulk-truncated substrate periodicity; $(0,1)_{\mathrm{rect}}$ (black) is the projection of (0,1) onto the axis of the rectangular superstructure. The glide-plane symmetry results in extinction of odd fractional spots along the axes of the rectangular cell (blue dashed circles). The anti-phase domain boundaries cause spot-splitting in the FFT, indicated with pairs of red arrows. (**C**) Constant-height nc-AFM image of the three rotational domains rotated by 120°. The image was obtained at 10 K, with an oscillation amplitude of 200 pm and 0 V sample bias. The image in (C) was acquired at a larger tip-sample distance than (A).

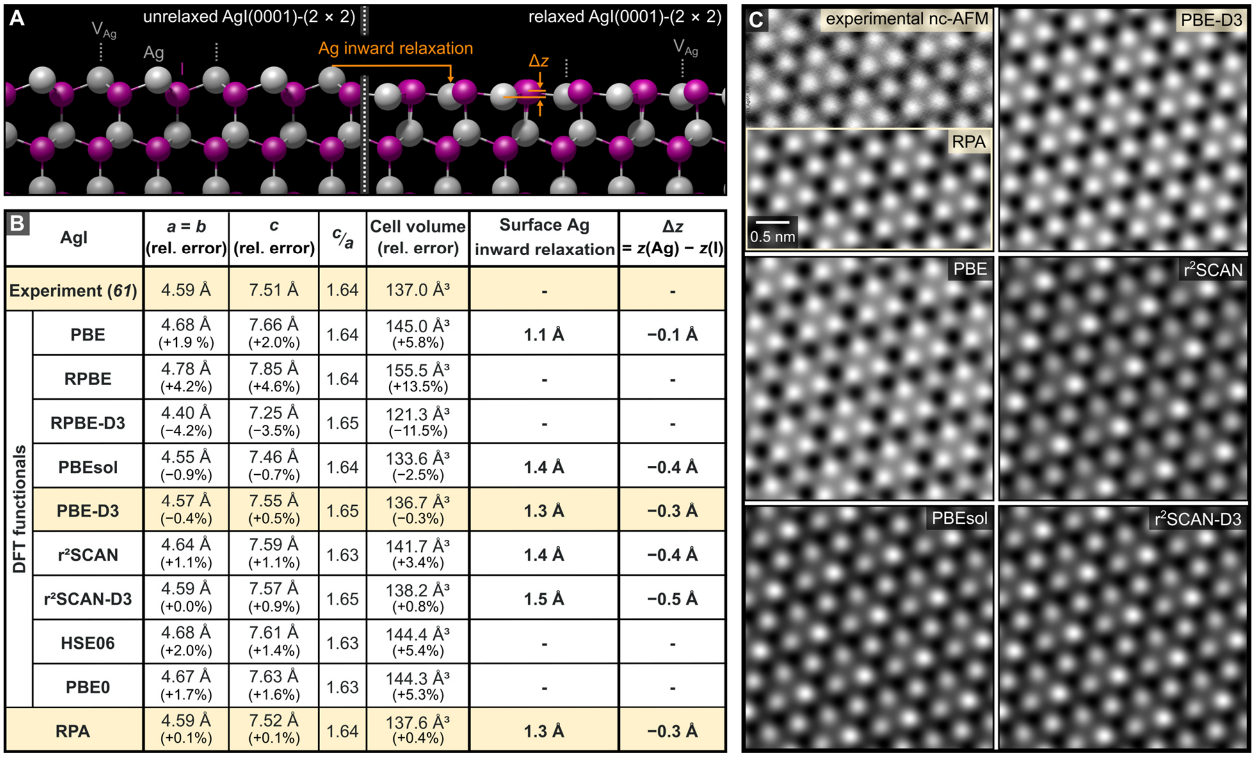

B

	AgI	$a = b$ (rel. error)	c (rel. error)	c/a	Cell volume (rel. error)	Surface Ag inward relaxation	Δz = z(Ag) − z(I)
	**Experiment (*61*)**	4.59 Å	7.51 Å	1.64	137.0 Å³	-	-
DFT functionals	**PBE**	4.68 Å (+1.9 %)	7.66 Å (+2.0%)	1.64	145.0 Å³ (+5.8%)	**1.1 Å**	**−0.1 Å**
DFT functionals	**RPBE**	4.78 Å (+4.2%)	7.85 Å (+4.6%)	1.64	155.5 Å³ (+13.5%)	-	-
DFT functionals	**RPBE-D3**	4.40 Å (−4.2%)	7.25 Å (−3.5%)	1.65	121.3 Å³ (−11.5%)	-	-
DFT functionals	**PBEsol**	4.55 Å (−0.9%)	7.46 Å (−0.7%)	1.64	133.6 Å³ (−2.5%)	**1.4 Å**	**−0.4 Å**
DFT functionals	**PBE-D3**	4.57 Å (−0.4%)	7.55 Å (+0.5%)	1.65	136.7 Å³ (−0.3%)	**1.3 Å**	**−0.3 Å**
DFT functionals	**r²SCAN**	4.64 Å (+1.1%)	7.59 Å (+1.1%)	1.63	141.7 Å³ (+3.4%)	**1.4 Å**	**−0.4 Å**
DFT functionals	**r²SCAN-D3**	4.59 Å (+0.0%)	7.57 Å (+0.9%)	1.65	138.2 Å³ (+0.8%)	**1.5 Å**	**−0.5 Å**
DFT functionals	**HSE06**	4.68 Å (+2.0%)	7.61 Å (+1.4%)	1.63	144.4 Å³ (+5.4%)	-	-
DFT functionals	**PBE0**	4.67 Å (+1.7%)	7.63 Å (+1.6%)	1.63	144.3 Å³ (+5.3%)	-	-
	**RPA**	4.59 Å (+0.1%)	7.52 Å (+0.1%)	1.64	137.6 Å³ (+0.4%)	**1.3 Å**	**−0.3 Å**

Fig. S5. Comparison of computational approaches to modeling AgI. (**A**) Inward relaxation of surface Ag atoms from bulk positions to relaxed positions on the (2 × 2)-reconstructed AgI(0001) surface. The parameter Δ*z* represents the height difference of relaxed Ag atoms relative to the surface I plane. Positions of Ag vacancies are indicated with gray dotted lines. (**B**) Bulk unit cell parameters of AgI obtained using different DFT functionals and RPA calculations, compared with experimental data (*53*). RPA provides the best agreement with experiment. Among the DFT functionals tested (each with its optimized lattice constants), PBE-D3 gives the closest match to RPA for both bulk lattice parameters and surface relaxation. Functionals deviating by > 1.4% from experimental lattice constants (RPBE, RPBE-D3, HSE06, and PBE0) were excluded from surface structure optimization. (**C**) Simulated nc-AFM images of models computationally optimized using the methods in (B). All images were generated at a constant tip-sample distance, accounting for differences in surface relaxation. AFM simulations based on RPA- and PBE-D3-relaxed structures closely reproduce the experimental image, whereas other functionals produce non-uniform relaxations of surface atoms, causing uneven contrast (PBE, I atoms adjacent to Ag vacancies appear brighter than I atoms surrounded by three Ag atoms), and disrupting the threefold symmetry of the reconstructed surface (PBEsol, r^{2}SCAN, and r^{2}SCAN-D3).

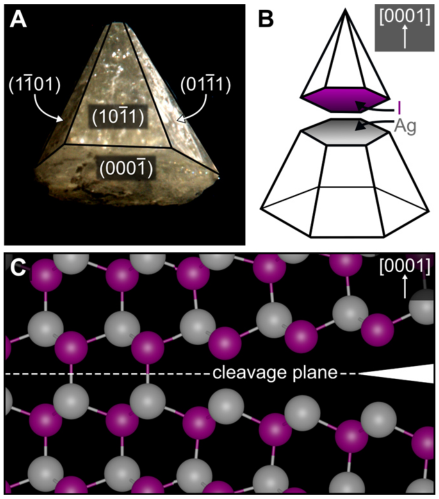

Fig. S6. Orientation of AgI crystals. Single-crystal X-ray diffraction (SC-XRD) analysis confirms that the AgI(0001) surface is Ag-terminated, while the AgI(000$\bar{1}$) is I-terminated. (**A**) Photograph of an AgI crystal with indexed faces. (**B**) Schematic illustration of the basal Ag- and I-terminated planes. (**C**) Structure model of β-AgI showing the cleavage plane; Ag atoms are shown in gray, I atoms in purple.

Data S1. (separate file)
Structure model of the reconstructed AgI(0001) surface in CIF format.

Data S2. (separate file)
Structure model of the epitaxial ice layer on the reconstructed AgI(0001) surface in CIF format.

Data S3. (separate file)
SC-XRD cif structure file.

checkCIF/PLATON report

Structure factors have been supplied for datablock(s) AgI

THIS REPORT IS FOR GUIDANCE ONLY. IF USED AS PART OF A REVIEW PROCEDURE FOR PUBLICATION, IT SHOULD NOT REPLACE THE EXPERTISE OF AN EXPERIENCED CRYSTALLOGRAPHIC REFEREE.

No syntax errors found.   CIF dictionary   Interpreting this report

Datablock: AgI

Bond precision:	I-Ag = 0.0003 A		Wavelength=0.71073
Cell:	a=4.59637(17)	b=4.59637(17)	c=7.5176(3)
	alpha=90	beta=90	gamma=120
Temperature:	292 K		

	Calculated	Reported
Volume	137.543(12)	137.544(9)
Space group	P 63 m c	P 63 m c
Hall group	P 6c -2c	P 6c -2c
Moiety formula	Ag I	Ag I
Sum formula	Ag I	Ag I
Mr	234.77	234.77
Dx,g cm-3	5.669	5.669
Z	2	2
Mu (mm-1)	18.162	18.160
F000	200.0	197.5
F000’	197.34	
h,k,lmax	8,8,13	7,4,13
Nref	343[182]	343
Tmin,Tmax	0.015,0.022	0.074,0.343
Tmin’	0.008	

Correction method= # Reported T Limits: Tmin=0.074 Tmax=0.343
AbsCorr = GAUSSIAN

Data completeness= 1.88/1.00   Theta(max)= 39.020

R(reflections)= 0.0397(329)   wR2(reflections)= 0.1011(343)

S = 0.937   Npar= 8

The following ALERTS were generated. Each ALERT has the format
test-name_ALERT_alert-type_alert-level.
Click on the hyperlinks for more details of the test.

Alert level C

```
PLAT923_ALERT_1_C S    Values in the CIF and FCF Differ by .......     -0.032 Check
PLAT973_ALERT_2_C Check Calcd Positive Resid. Density on       Ag1       1.21 eA-3
```

Alert level G

```
PLAT004_ALERT_5_G Polymeric Structure Found with Maximum Dimension          3 Info
PLAT068_ALERT_1_G Reported F000 Differs from Calcd (or Missing)...     Please Check
PLAT152_ALERT_1_G The Supplied and Calc. Volume s.u. Differ by ...          3 Units
PLAT769_ALERT_4_G CIF Embedded Explicitly Supplied Scattering Data     Please Note
PLAT969_ALERT_5_G The 'Henn et al.' R-Factor-gap value ..........      7.442 Note
              Predicted wR2: Based on SigI**2  1.35 or SHELX Weight 10.39
PLAT982_ALERT_1_G The Ag-f'=   -0.8749 Deviates from  IT-Value =      -0.8971 Check
PLAT982_ALERT_1_G The  I-f'=   -0.4378 Deviates from  IT-Value =      -0.4742 Check
PLAT983_ALERT_1_G The Ag-f"=    1.1063 Deviates from  IT-Value =       1.1015 Check
PLAT983_ALERT_1_G The  I-f"=    1.8209 Deviates from  IT-Value =       1.8119 Check
```

0 **ALERT level A** = Most likely a serious problem - resolve or explain
0 **ALERT level B** = A potentially serious problem, consider carefully
2 **ALERT level C** = Check. Ensure it is not caused by an omission or oversight
9 **ALERT level G** = General information/check it is not something unexpected

7 ALERT type 1 CIF construction/syntax error, inconsistent or missing data
1 ALERT type 2 Indicator that the structure model may be wrong or deficient
0 ALERT type 3 Indicator that the structure quality may be low
1 ALERT type 4 Improvement, methodology, query or suggestion
2 ALERT type 5 Informative message, check

It is advisable to attempt to resolve as many as possible of the alerts in all categories. Often the minor alerts point to easily fixed oversights, errors and omissions in your CIF or refinement strategy, so attention to these fine details can be worthwhile. In order to resolve some of the more serious problems it may be necessary to carry out additional measurements or structure refinements. However, the purpose of your study may justify the reported deviations and the more serious of these should normally be commented upon in the discussion or experimental section of a paper or in the "special_details" fields of the CIF. checkCIF was carefully designed to identify outliers and unusual parameters, but every test has its limitations and alerts that are not important in a particular case may appear. Conversely, the absence of alerts does not guarantee there are no aspects of the results needing attention. It is up to the individual to critically assess their own results and, if necessary, seek expert advice.

Publication of your CIF in IUCr journals

A basic structural check has been run on your CIF. These basic checks will be run on all CIFs submitted for publication in IUCr journals (*Acta Crystallographica*, *Journal of Applied Crystallography*, *Journal of Synchrotron Radiation*); however, if you intend to submit to *Acta Crystallographica Section C* or *E* or *IUCrData*, you should make sure that full publication checks are run on the final version of your CIF prior to submission.

Publication of your CIF in other journals

Please refer to the *Notes for Authors* of the relevant journal for any special instructions relating to CIF submission.

PLATON version of 02/02/2025; check.def file version of 02/02/2025

Datablock AgI - ellipsoid plot

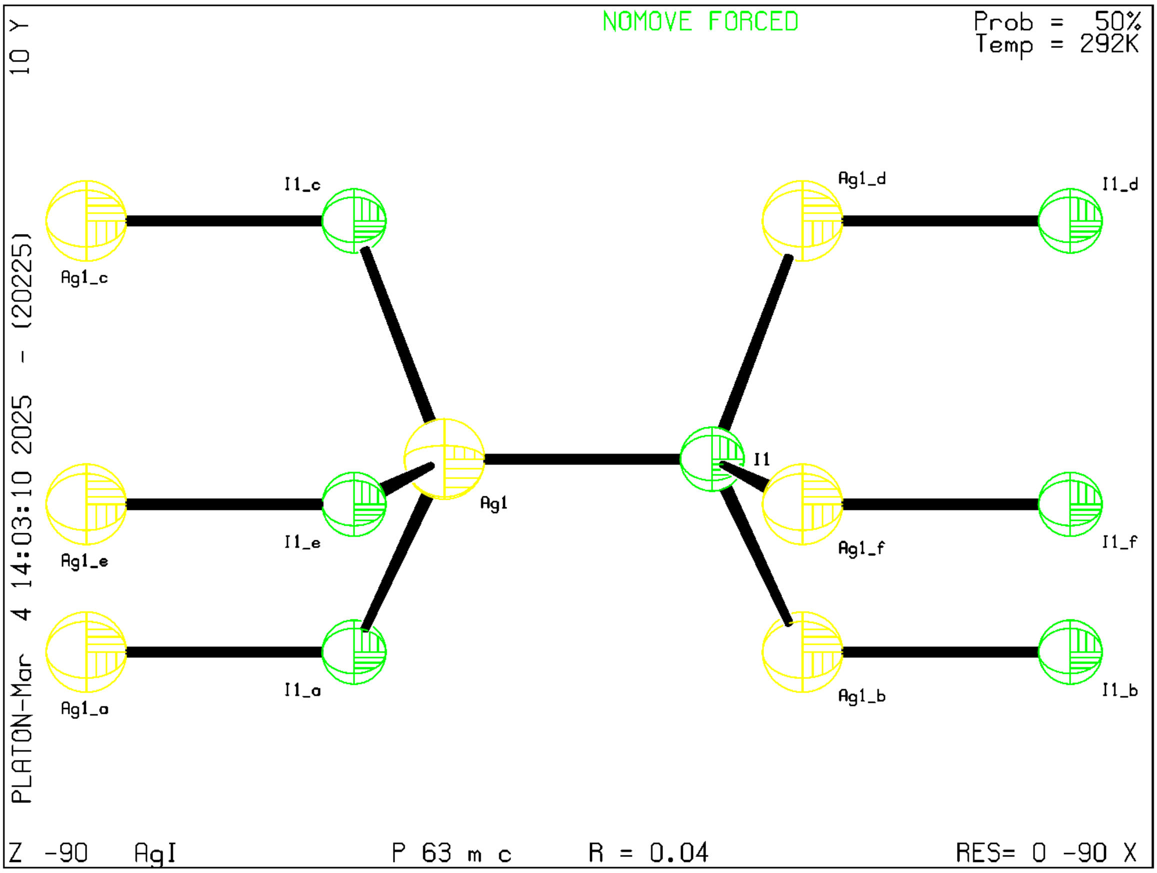

Supplement: Supplementary file 1 — Figs. S1 to S6 Legends for data S1 to S3 SC-XRD checkCIF/PLATON report [file sciadv.aea2378_sm.pdf]
